# A Little Bit of Heart

**DOI:** 10.31486/toj.21.5017

**Published:** 2021

**Authors:** Ronald G. Amedee

**Affiliations:** Head of Clinical School and Professor, The University of Queensland Faculty of Medicine, Ochsner Clinical School, New Orleans, LA; Editor-in-Chief, *Ochsner Journal*

Some old-fashioned things like fresh air and sunshine are hard to beat.

–Laura Ingalls Wilder

This issue of the *Ochsner Journal* contains general medical topics articles that should prove interesting and educational for our readers. Six original research articles, two quality improvement papers, and one review/contemporary update serve as the foundation pieces for this edition.

Regarding original research, “Use of Web-Based Patient Portals in Patients With Atrial Fibrillation Is Associated with Higher Readmissions” by Davis, Wilson, Erwin, et al is a particularly enlightening manuscript, as is “Perceptions of a Pragmatic Family-Centered Approach to Childhood Obesity Treatment” authored by Kennedy, Davison, Fowler, et al. “A Nurse-Driven Protocol for Foley Catheter Utilization Decreases the Incidence of Traumatic Foley Catheterization” by Laborde, Hill, Dukovac, et al highlights a patient safety project successfully advanced by our own urology department at Ochsner Health.

“Building a Culture of Well-Being in Primary Care Resident Training Programs” authored by Stansfield, Kenaga, and Markova describes a quality improvement project conducted by a member institution of the Alliance of Independent Academic Medical Centers for one of the Alliance's National Initiatives. Bazzano, Durant, and Brantley offer a contemporary review/update in “A Modern History of Informed Consent and the Role of Key Information.”

Eight case reports are also included in this issue, including “Gastroduodenal Artery Pseudoaneurysm Causing Obstructive Jaundice” by Chapman, Bolton, Gioe, et al and “Use of Patient-Specific 3-Dimensional Printed Models for Planning a Valve-in-Valve Transcatheter Aortic Valve Replacement and Educating Health Personnel, Patients, and Families” authored by Soto and Betancourt.

The Fox, Keflemariam, Cornett, et al case report “Structural Heart Issues in Dextrocardia: Situs Type Matters” imparts key clinical observations on the topic. Images from that case are on the cover of this issue. As you may have noticed, we have a bit of a heart theme going in this edition, along with a bit of an orthopedics theme. Cases from Ochsner Orthopedics explore the complex repair of a hand injury (“Closed Traumatic A2 Through A4 Pulley Rupture and Flexor Digitorum Superficialis Avulsion Treated With Reconstruction”) and the extremely rare congenital condition discoid medial meniscus (“Arthroscopic Saucerization of a Symptomatic Posterior Horn Tear in a Discoid Medial Meniscus”). In another orthopedics-related note, just about a year ago, Dr Andrew Gottschalk pitched the idea of including a quarterly sports medicine column in the *Journal* to explore topics of general interest to practitioners in multiple specialties, particularly primary care physicians who are often the first clinicians to see patients with these conditions. The column has definitely enhanced the *Journal*, and we have acknowledged Dr Gottschalk's contributions by naming him featured contributor for the spring issue. You can see his photo and read his bio at the *Ochsner Journal* website (www.ochsnerjournal.org).

As I am writing this introduction to the spring issue, Louisiana has successfully administered more than 1 million COVID-19 vaccine injections, and the governor has announced a move to the more relaxed Phase 3 restrictions in our statewide recovery. A third approved vaccine (Johnson & Johnson) is now available and being distributed, and we collectively look forward to achieving herd immunity by late spring or early summer and hopefully returning to normalcy. Until then, remember just how hard we have struggled and how far we have come in our fight against this pandemic. Please stay safe and healthy.

